# Medical News

**Published:** 1856-09

**Authors:** 


### MEDICAL NEWS.

THE New York Journal of Medicine and the New York Medical Times have been combined, under the title of the New York Journal of Medicine. The Editors are Dr. S. S. Purple, S. Smith and H. D. Bulkley. The Times was an excellent and ever welcome Journal, and we shall greatly miss its disappearance from our list of exchanges.

—

MEDICAL DEPARTMENT OF PENNSYLVANIA COLLEGE.—The following resolutions, in relation to the resignation of Prof. Allen, were unanimously adopted by the Faculty, and ordered to be published:—

*Resolved,* That the Faculty of the Medical Department of Pennsylvania College sincerely regret that their colleague, Dr. J. M. Allen, has felt constrained to resign the Chair of Anatomy, which, for several years, he has filled with signal ability and distinction.

*Resolved,* That in accepting Dr. Allen's resignation, the Faculty desire to assure him of their high estimate of his professional talent, to reciprocate his expressions of friendly feeling, and to offer him their cordial good wishes for his success in whatever career he may determine to pursue.

FRANCIS G. SMITH, M. D.,
*Registrar pro. tem.*

A disciple of Dr. Coffin has been tried at Lincoln, and sentenced to three months' imprisonment, for manslaughter by the use of *Lobelia inflata.* The victim in this case was the wife of a laboring man, and was said to be suffering from fever, but was in a convalescent state; when the quack doctor introduced himself to her, he pronounced that she had "abscess in her inside," and that he would cure her. He accordingly administered the panacea, which was taken so regularly and so effectually, that, after great suffering and delirium, the poor woman died. The cases of poisoning by lobelia inflata have been so numerous of late years, that this drug ought certainly to be classed among the prohibited articles, if any legislation should take place upon the sale of poisons. In the case to which we now allude, the punishment of imprisonment for three months appears a very light one; but it has been so frequently the case that prisoners charged with poisoning by lobelia inflata have been triumphantly acquitted (sometimes under the direction of the presiding judge), that the mere fact of a conviction is a subject for congratulation.—*London Med. Times and Gaz., July,* 1856.

---

In a former communication I stated my wish to bring under your notice the fatal case of small-pox, the result of inoculation with variolous matter, which occurred some time since in the neighborhood of Ballinasloe. It is lamentable to see the obstinacy with which the lower classes adhere to this most baneful practice, and their blindness in allowing themselves to be made the dupes, and their children the victims, of the ignorant and unprincipled individuals who infest the country in pursuit of their unholy traffic. It is also humiliating to observe how little effect legislative enactment has in checking this monstrous evil, chiefly in consequence of the great unwillingness of the people to give evidence against the delinquents. An eminent practitioner writes, "Small-pox inoculations have been extensively practised in this town and neighborhood. Though we had sufficient moral evidence of this, we were not able to prove its legality in any case previously to those referred to in the newspapers. The culprit in the late instance had, on pleading guilty of one offence, been sentenced to a fortnight's imprisonment, and, subsequently to the sentence, information was received of a case which had terminated fatally. The resident magistrate, with laudable determination, insisted on a post-mortem examination being held, and the coroner's inquest, which had been summoned, was adjourned until the autopsy should be performed. The result was a verdict of manslaughter. The consequences of this practice of inoculation have been very disastrous. Variola rages in this town with great violence, in some instances attacking the upper classes of society, and I trust that something useful may result from the verdict which has been obtained." The evil described in the foregoing communication has evidently attained a fearful height, and loudly calls for effective interference. It appears to me that the statute bearing on the point is far too lenient; I can find in force in Ireland on the subject, only the 3d and 4th Victoria, c. 29, § 8, which provides that persons inoculating, or otherwise producing small-pox, shall be liable to be proceeded against at petty sessions, and, on summary conviction before one or two magistrates, may be imprisoned for a period not exceeding one month.—*Dublin Correspondent of London Med. Times and Gaz.*

---

Dr. Valentine Mott.—In an interesting paper by Dr. Stewart in this month's *Edinburgh Journal*, describing the condition of the Medical Profession in the United States, he thus enumerates the successful career pursued by Dr. Valentine Mott as an operative surgeon:—"The whole world accords him the credit due to his first and greatest undertaking—the ligature of the arteria innominata. The patient, whose primitive iliac he tied more than twenty-five years ago, still lives in the enjoyment of perfect health. He has ligatured the external iliac artery seven times, and lost three of the patients; the internal twice, both of whom were cured; the femoral artery fifty-two times, with the loss of one patient; the primitive carotid forty-two times, with a fatal result in two instances; the subclavian, *without* the scaleni, six times, all successful; and *within* once, the patient dying of secondary hæmorrhage. His original operations upon the maxillary bones have been very numerous; and he considers his exsection of the clavicle for osteo-sarcoma as the most difficult and dangerous operation that can be undertaken. He has cut for the stone 164 times, with a loss of seven patients. His operative career, though he is nearly 70 years of age, has not yet terminated, for he writes word that his second case of ligature of the internal iliac has just left the city."—*Med. Times and Gaz.*

---

Deaths in the French Army.—The French Government has just published an account of the number of deaths that occurred in the Army of the East between May 1, 1854, when the embarkation for Turkey commenced, to March 30, 1856, when the Treaty of Peace was signed:

| | |
|---|---|
| Officers of all ranks, - - - - - - | 1284 |
| Non-commissioned officers, - - - - | 4403 |
| Soldiers, - - - - - - - - - | 56,805 |
| Total - - - - - - - - - | 62,492 |

The following are the returns of deaths of all ranks and arms which occurred during the above two years in the entire French army, not in the Crimea:—

| | |
|---|---|
| In Algeria, - - - - - - - - - | 5246 |
| In Italy, - - - - - - - - - | 1088 |
| Baltic Expedition, - - - - - - - | 1059 |
| In France, - - - - - - - - - - | 13,636 |
| Total - - - - - - - | 21,028 |

As the army in the Crimea was never more than about a fifth of the entire French army, the mortality during the Crimean campaign has been indeed enormous. Many single regiments lost more than 1000 men. Thus, the 7th Infantry lost 1662; the 26th, 1511; the 28th, 1505; the 98th, 1471, etc.—[We have reason to believe that the mortality was in reality much greater.—Ed. *London Med. Times and Gaz.*]

The French Hospitals in the East.—The great hospital at Pera has recently been closed, owing to the return to France of a portion of the French army. There were at one time as many as 2000 patients in this hospital, and no less than 27,500 were received during the two months it remained open. Out of this number, 13,000 were sent to France or to other hospitals in a convalescent state. The *Gazette Médicale de Paris*, in recording these figures, does not say whether the remaining 14,500 died; such, however, might perhaps be the inference. The total number of days' treatment was 633,985.—*London Lancet.*

---

In the March number, 1856, of the Examiner, we published a translation of a paper, entitled, "Results of some Statistical and Physiological Researches on Twins;" at the end of which, the Translator added some remarks, giving the results of *his own* investigations of the subject gathered from the published statistics of the Dublin Lying-in Hospital. In the July number of the Glasgow Medical Journal, we find the *whole* article copied, not only without the customary credit, but with the following introductory:—"At a meeting of the French Academy of Sciences, November 25, Mr. Baillarger read a paper containing the results of some Statistical and Physiological Researches on Twins. We have thought the subject presents some features which might interest the readers of the *Journal.*" Precisely the same words introduced the paper in our own Journal, excepting that the word "Examiner" is used in place of the "Journal." From the change of word, it would almost seem as if "the Journal" claimed the translation of the paper for itself. We are willing to believe, however, that the omission of the usual courtesy was an oversight, and we take this opportunity of drawing the Editor's attention to it.

We not unfrequently notice our own articles thus appropriated by other Journals. A little more attention to the rules of courtesy and the rights of others would prevent such mistakes, which are disagreeable to both parties.

